# Occupational therapy addressing the ability to perform activities of daily living among persons living with chronic conditions: a randomised controlled pilot study of ABLE 2.0

**DOI:** 10.1186/s40814-021-00861-9

**Published:** 2021-06-11

**Authors:** Vita Hagelskjær, Kristina Tomra Nielsen, Cecilie von Bülow, Maud Graff, Eva Ejlersen Wæhrens

**Affiliations:** 1 The Parker Institute, Bispebjerg and Frederiksberg Hospital, University of Copenhagen, Copenhagen, Denmark; 2 Occupational Science & Occupational Therapy, User Perspectives and Community-based Interventions, Department of Public Health, University of Southern Denmark, Odense, Denmark; 3 Department of Occupational Therapy, VIA University College, Holstebro, Denmark; 4 Department of Occupational Therapy, University College of Northern Denmark, Aalborg, Denmark; 5 Department of Rehabilitation & Scientific Institute for Quality of Care Research, Radboud University Medical Center, Nijmegen, The Netherlands

**Keywords:** ADL ability, Everyday life, Goal setting, Complex interventions, Occupational Therapy Intervention Process Model (OTIPM), Rehabilitation

## Abstract

**Background:**

The ABLE intervention was developed to enhance the ability to perform activities of daily living (ADL) tasks among persons living with chronic conditions. ABLE is a generic, home-based, individualised, 8-week occupational therapy intervention program, developed to be delivered in Danish municipalities. In a previous study, the feasibility of ABLE was evaluated in terms of content and delivery. In this pilot study, the remaining feasibility aspects of a randomised controlled trial including (i) trial procedures (recruitment and retention), (ii) randomisation, (iii) adherence to program, (iv) feasibility of additional outcome measurements, and (iv) access to information on usual occupational therapy were evaluated.

**Methods:**

The study was conducted in a Danish municipality, using a two-armed parallel randomised controlled design, planning a recruitment strategy including 20 persons living with one/more chronic conditions and experiencing problems performing ADL. The following progression criteria were used to determine if a future full-scale randomised controlled trial was feasible: (i) recruitment (50% met the eligibility criteria) and retention (80%), (ii) randomisation (80% accepted randomisation, procedure was executed as planned), (iii) adherence to program (100% followed the treatment protocol), (iv) outcome measurements (80% of the participants delivered relevantly and fully answered questionnaires), and (v) usual occupational therapy (extraction of needed information was successful).

**Results:**

Due to the COVID-19 pandemic, the study was truncated resulting in limited but sufficient data to answer most of the study questions. (i) Eighteen of 37 eligible persons (48.6%) were recruited; of those treated (*n* = 6), all remained (100%); (ii) 18 accepted randomisation (100%), and procedure was effective; (iii) ABLE was delivered with adherence (100%); (iv) 92.3–100% of the participants gave relevant and complete answers in two of three questionnaires; and (v) needed information on usual occupational therapy was extractable in seven of nine aspects.

**Conclusions:**

Proceeding to full-scale trial is recommendable; however, a few adjustments on outcome measurements, inclusion criteria and extraction of information on usual occupational therapy are needed.

**Trial registration:**

The study was registered at ClinicalTrials.gov (Identifier: NCT04295837) on December 5th, 2019. Retrospectively registered.

**Supplementary Information:**

The online version contains supplementary material available at 10.1186/s40814-021-00861-9.

## Key messages regarding feasibility

- *What uncertainties existed regarding the feasibility*? The development of the ABLE intervention program and feasibility aspects related to content and delivery have been addressed in previous studies. This pilot study addressed remaining uncertainties including evaluation of trial procedures (recruitment and randomisation), adherence, access to information on usual occupational therapy and feasibility of additional outcome measurements.
- *What are the key feasibility findings*? The procedures for recruitment and randomisation were feasible; the ABLE intervention program was adherently delivered; and almost all the desired information on usual occupational therapy was accessible. In terms of the feasibility of outcome measurements, the administration of the ADL-Questionnaire (ADL-Q) in this client population was associated with challenges, whereas the Occupational Balance Questionnaire (OBQ11) and Client Weighted Problems questionnaire (CWP) were appropriate.
- *What are the implications of the feasibility findings for the design of the main study*? The study results implied a need for a few adjustments related to inclusion criteria, extraction of information on usual occupational therapy and to the outcome measurements. A full-scale randomised controlled trial is recommended.

## Background

The number of persons living with chronic conditions is increasing worldwide. A recent register-based study [1] has revealed that 65.5% of Danish residents, aged 16 or above, have one or more chronic condition. Several studies provide evidence to support that persons with chronic conditions generally experience problems performing activities of daily living (ADL) tasks [2–9]. This is also reflected in the definition of chronic conditions proposed by Goodman et al.: ‘C*onditions that last 1 year or more and require ongoing medical attention and/or limit activities of daily living*’ [10]. ADL involve tasks that most people need to perform in their everyday lives, including personal and instrumental ADL tasks [11]. Personal ADL involve basic self-care tasks that are necessary to perform for all people across gender, age, culture and interests. Examples are eating, toileting, grooming and dressing. Instrumental ADL tasks involve more complex household chores, necessary for independent living, including shopping, cooking, cleaning and doing laundry [12]. Addressing ADL task performance problems is a core element in occupational therapy and results from studies indicate that occupational therapy interventions in general may improve ADL ability among older persons with various chronic conditions [13–16]. However, rigorous studies, testing the outcomes of occupational therapy for persons living with chronic conditions experiencing ADL task performance problems are limited [13–17]. A scoping review on occupational therapy for chronic conditions [14] suggested that similar interventions addressing ADL may be applicable across a range of diagnoses. In support of this, a study examining self-reported quality of ADL task performance among *n* = 593 persons living with chronic conditions [18, 19], found similar types of ADL task performance problems across a range of chronic conditions. Hence, there was a need to develop a generic intervention program to address decreased ADL ability across chronic conditions causing disability.

Accordingly, the research program “A better everyday life”, launched in 2015, aims to develop and evaluate an occupational therapy intervention program (named ABLE) focusing on enhancing the ADL ability among persons living with chronic conditions experiencing ADL task performance problems. The research program is guided by the British Medical Research Council’s (MRC) guidance on how to develop and evaluate complex interventions [20]. The guidance prescribes four stages: (1) Development, (2) Feasibility/piloting, (3) Evaluation, and (4) Implementation [20].

In prior phases, the first version of the ABLE intervention program (ABLE 1.0) was developed [18, 19, 21] incorporating knowledge based on existing evidence, clinical expertise of occupational therapists (OTs) and client needs [18, 19, 21, 22]. Moreover, feasibility was addressed in terms of content and delivery, and the selected outcome measurements were ascertained [23]. However, the feasibility evaluation also revealed a need to adjust the recruitment procedure, apply minor changes to the intervention manual and further monitor adherence to the intervention program [23]. A pilot randomised controlled trial (RCT) study was recommended before proceeding to a full-scale RCT [23].

Consequently, the ABLE intervention program faced pilot testing involving evaluation of trial procedures (including recruitment and randomisation), adherence, feasibility of additional outcome measurements and access to information on usual occupational therapy. In preparation for this, a new strategy for recruitment was planned, a randomisation procedure was developed, the ABLE intervention program manual was revised (ABLE 2.0), questions related to evaluating the feasibility of additional outcome measurements were developed and decisions regarding needed information on usual occupational therapy were made.

## Methods

### Aims and objectives

The overall aim of this ABLE pilot study was to inform the decision on whether to proceed to full-scale RCT. The pilot study should strengthen the design and conduct a future RCT, in relation to the remaining feasibility aspects [24, 25]. The specific aims of the ABLE pilot study were to:

(i) Assess effectiveness of the recruitment process and retention in the context of a future trial
(ii) Assess the randomisation procedure and determine the acceptability of randomisation among the participants
(iii) Assess adherence to intervention program in the same context as the future RCT trial
(iv) Assess appropriateness of additional outcome measurements
(v) Determine if needed information on usual occupational therapy can be extracted from the client records in the municipality

### Study design

The ABLE pilot was designed as a two-armed parallel randomised controlled study with random and stratified allocation to ABLE 2.0 and usual occupational therapy, respectively (*n* = 20).

### Setting, participants and recruitment

The ABLE pilot study was scheduled to be conducted from January to May 2020 in a Danish municipality, with almost 90,000 inhabitants. The Rehabilitation Unit in the municipality is organised in four comparable geographic areas (North, East, South, and West). Participants were recruited from all four areas. ABLE 2.0 and usual occupational therapy sessions were delivered, and data collection was conducted, in the homes of the participants. Participants in both the ABLE intervention group and the control group (usual occupational therapy) received other health care services as usual.

Eligible participants lived with one or more medically diagnosed chronic condition, were aged ≥ 18 years; lived in own home; experienced ADL task performance problems; communicated independently and relevantly (without severe cognitive deficits); were motivated and ready for making changes in performance of ADL; motivated and ready for cooperating with an occupational therapist (OT) in finding solutions to the experienced problems; and able to understand and relevantly answer a questionnaire. Exclusion criteria were known substance abuse; mental illness and/or other acute illness effecting ADL task performance; or language barriers.

OTs delivering ABLE 2.0 (ABLE OTs) (*n* = 3) were recruited among OTs in the municipality based on having at least 2 years of experience working with persons living with chronic conditions and ADL task performance problems, and being calibrated as Assessment of Motor and Process Skills (AMPS) [26, 27] raters. In preparation for delivering ABLE 2.0, the ABLE OTs participated in a three-and-a-half-day tailored workshop.

Assessors (*n* = 2), conducting observation-based outcomes evaluation of ADL ability using the AMPS at baseline and post-intervention, were OTs trained and calibrated as AMPS raters recruited from a nearby Hospital Unit.

Participants were recruited using a two-step model. In the first step, all persons referred to rehabilitation services in the municipality, or persons already receiving any kind of rehabilitation services, were screened for eligibility. A key OT in each Rehabilitation Unit area performed the screening based on a guideline including a checklist on the eligibility criteria. In a telephone conversation, the key OT provided the potential participant with initial information on the ABLE pilot study and asked for permission to forward contact information to the primary investigator. In the second step, and within three weekdays from the forwarded contact information, the primary investigator called to provide more detailed information, determine if the person was interested in participating and finalise screening of eligibility for inclusion. If the person met the inclusion criteria, preliminary oral consent to participate was obtained.

### ABLE 2.0 intervention program

**Fig. 1:**
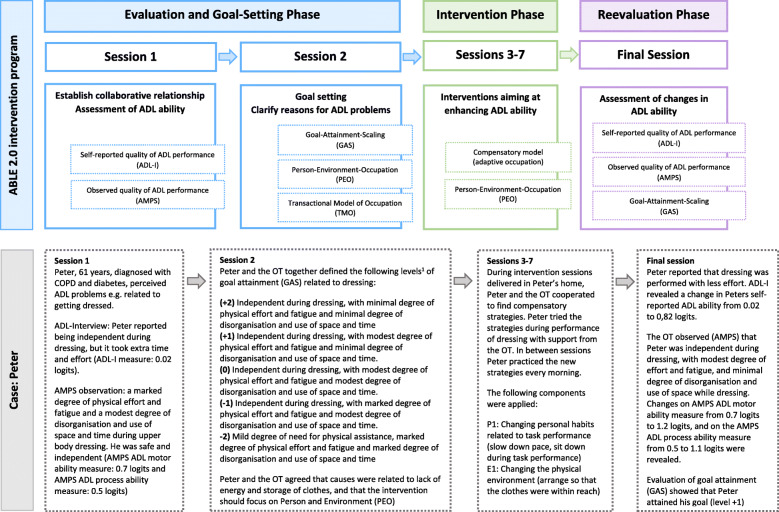
The ABLE 2.0 Intervention Program including a brief case example. ^1^ GAS levels of scoring: The level of goal attainment is described using an ordinal scale from −2 to +2. The actual level of performance is described at level −1, and the expected level is described at level 0. Levels +1 and +2 are descriptions of what the person will be able to, if he or she achieves more than expected. Level −2 describes the level, where the person achieves less than expected. ADL=Activities of Daily Living; ADL-I= Activities of Daily Living-Interview; AMPS=Assessment of Motor and Process Skills; COPD= Chronic Obstructive Pulmonary Disease; GAS=Goal Attainment Scaling, OT=Occupational Therapist; PEO=Person Environment Occupation; TMO=Transactional Model of Occupation

The manualised ABLE 2.0 intervention program is a generic, systematic and client-centred 8-week occupational therapy intervention program, addressing ADL task performance problems among persons living with chronic conditions. It is characterised by offering an individualised combination of intervention components adapted to the single person. Three models underpin the ABLE intervention program, namely the Occupational Therapy Intervention Process Model (OTIPM) [28], describing the problem-solving process (here using an adaptational approach); the Person-Environment-Occupation (PEO) model [29], here explaining performance of daily activities as doing shaped by the interaction between person, environment and occupation; and finally, the Transactional Model of Occupation (TMO) [28], clarifying reasons for ADL task performance problems. ABLE 2.0 consists of five to eight sessions (Fig. 1).

Session 1 involves standardised assessment of perceived and observed ADL ability by means of the ADL-Interview (ADL-I) [30] and the Assessment of Motor and Process Skills (AMPS) [26, 27], respectively. AMPS is an observation-based assessment measuring two aspects of ADL task performance: ADL motor ability (reflecting physical effort) and ADL process ability (reflecting efficiency, safety and independence) [26, 27].

Session 2 concerns setting client-centred goals using Goal Attainment Scaling (GAS) [31, 32] based on ADL task performance problems identified and prioritised using ADL-I in session 1.

Sessions 3–7 are intervention sessions, focusing on adaptation by employing a combination of intervention components to improve ADL task performance, and delivered face-to-face or by telephone, with or without homework (e.g. practicing strategies and trying out new ways of doing) between sessions.

The final session includes re-evaluation of self-reported and observed ADL ability using the ADL-I and AMPS and evaluation of goal attainment using GAS.

Mandatory sessions are 1–2, and at least two intervention sessions and the final session. The program is delivered in the homes or local areas of the participants and is designed to be implemented as part of community-based rehabilitation. Structure and overall content of ABLE 2.0, including a brief case example, is presented in Fig. 1; Table 2 provides information on the intervention components; and Additional file 1 provides description of instruments and tools used in the ABLE 2.0.

### Usual occupational therapy

Participants in the control group received standard occupational therapy services provided by the municipality (usual occupational therapy). To gain a preliminary insight into the usual occupational therapy prior to the pilot study, information was extracted from ten records representing people similar to those to be recruited for the pilot study. Those data suggested that the typical dose of usual occupational therapy was 3 x 60 min. Also, the content of usual occupational therapy seemed to vary based on client conditions and needs, but observation of ADL task performance, counselling and evaluation of the use of helping aids were common. Examples of established goals included “ability to bath independently” or “toilet safety”.

### Data collection

Criteria for progressing to full-scale RCT, based on data derived from this pilot study, were clarified using frameworks by O’Cathain [24], Bowen [33] and Charlesworth [34] and included the following aspects: Recruitment and retention, randomisation procedure, adherence to program, appropriateness of outcome measurements, and information on usual occupational therapy.

### Recruitment and retention

The previous feasibility study [23] revealed recruitment and retention challenges. Thus, 33% of the participants dropped out due to lack of motivation or reporting no need of intervention. Consequently, recruitment procedures in this pilot study were specified to recruit participants that actually experienced ADL task performance problems and were ready to make changes, using a two-step model, described above. Moreover, the former inclusion criteria on motivation and readiness for change were specified by splitting it into two criteria: (a) ‘*motivated and ready for making changes in performance of ADL’* and (b) ‘*motivated and ready for cooperating with OT in finding solutions to the experienced problems’.*

To monitor recruitment and retention, the flow of participants was registered, capturing information on (1) how many persons were contacted to recruit 20 participants for the pilot study, (2) reasons for accepting/not accepting to participate and (3) number of and reasons for dropouts. Progression criteria on recruitment were that 50% of the persons contacted met the eligibility criteria and accepted participation and that 80% stayed in the program.

### Randomisation procedure

To assess the randomisation procedure and determine the acceptability of randomisation among the participants, a randomisation procedure was developed reflecting a procedure to be employed in a future randomised controlled trial. Hence, before inclusion of participants, a randomisation list was generated based on permuted random blocks of variable size (2 to 6 in each block). Participants were allocated in a 1:1 ratio to either ABLE or usual occupational therapy taking into account baseline level of observed ADL ability, using AMPS ADL motor ability (≤ 1.0 vs > 1.0) and ADL process ability (≤ 0.7 vs > 0.7) independence cut-offs [26]. Potential participants were informed about randomisation procedures and given the possibility to withdraw. Monitoring method was to register the randomisation progress including reasons to refuse randomisation. Progression criteria were that 80% accepted randomisation and that procedures were executed as planned.

### Adherent delivery of ABLE 2.0

#### Changes to the ABLE manual

The previous feasibility study [23] revealed some deviations from the manual in delivery (e.g. omission of AMPS in first and/or final sessions, omission of goal setting due to participants having no goals to address, and delivery of less than the minimum of five sessions). Thus, steps were taken to increase adherence to the ABLE manual. The manual was revised, applying results from the feasibility study and also incorporating updates of the theoretical framework OTIPM [28] underpinning the intervention. To examine the revised ABLE manual in terms of any aspect that could lead to confusion or misunderstanding among OTs delivering the program, a cognitive debriefing [35] was conducted. The input and suggestions from the participating OTs (*n* = 5) were incorporated in the manual, resulting in ABLE 2.0. Further details on the cognitive debriefing process will be published in a separate paper.

#### Changes in training workshop

The training workshop for the ABLE OTs was extended to three-and-a-half days over a period of a month and providing in-between feedback on e.g. the OTs’ use of instruments and delivery of sessions. The workshop consisted of introduction to ABLE 2.0 including underlying intervention theories, practicing the use of instruments, and training delivery of the ABLE intervention components. It was emphasised why both initial AMPS evaluation and goal setting are regarded core mechanisms of change in the program.

#### Changes in physical environments

The ABLE feasibility study [23] reported limited access to helping aids to try out and practice using. Accordingly, direct access to helping aids was ensured in the pilot study.

To monitor adherence, registration forms were filled in after each session by both participant and OT informing on perceived engagement; participant involvement, meaningfulness and satisfaction with intervention. Furthermore, OT registration forms informed on number of sessions delivered and time use in each session (dose). Also, what was delivered, including deviations from manual, goal setting and instruments applied for evaluation of ADL ability (fidelity); confidence in delivering the program; unintended side effects; and practical and/or organizational facilitators and barriers. Aspects related to confidence in delivering the program; involvement of participant; OT’s and participant’s engagement, meaningfulness and satisfaction with the program, were scored using Likert scales from 1 to 5; 1 = very low degree, 2 = low degree, 3 = fair degree, 4 = high degree and 5 = very high degree. Progression criterion on adherence was ABLE 2.0 delivered as intended in terms of dose and fidelity.

### Appropriateness of outcome measurements

Several outcome measurements planned for application in the full-scale ABLE RCT were already evaluated for appropriateness in the feasibility study [23], but some remained to be tested: ADL-Questionnaire (ADL-Q) [36], Occupational Balance Questionnaire (OBQ11) [37], and five questions specifically constructed for this study, named Client Weighted Problems questionnaire (CWP) (Additional file 2). Appropriateness was evaluated by counting the number of relevantly and fully answered ADL-Q, OBQ11 and CWP questionnaires at baseline and post-intervention. Progression criterion was 80% of the participants giving relevant and complete answers in questionnaires.

#### ADL-Questionnaire

ADL-Q is a standardised evaluation tool to describe and measure self-reported quality of ADL task performance [36], in terms of physical effort and/or fatigue, efficiency, safety and independence. The persons report their perceived ADL ability for each of 47 ADL tasks using seven response categories: (a) I perform the task independently without use of extra time or effort and without risk; (b) I perform the task independently, but I use helping aids; (c) I perform the task independently, but it takes me extra time; (d) I perform the task independently, but I use extra effort/get tired; (e) I perform the task independently, but there is a risk that I might injure myself; (f) I need assistance from someone but do participate; and (g) the task is performed by others for me—I cannot participate actively. The person is instructed to use more than one response category, if several apply to their performance of the specific ADL task (e.g. mark both c and d if they spend extra time and get tired). Finally, ratings for personal ADL tasks should be based on ADL task performances within the past 24 h and for instrumental ADL tasks within the past 7 days [36].

To create an overall linear measure of self-reported quality of ADL task performance (reported in log-odds probability units; logits), based on the Rasch measurement methods, the mark given in the lowest response category on each task is re-scored using an ordinal rating scale from 0 to 3: C*ompetent* (score = 3) covering response categories (a) and (b), *Using extra time/effort* (score = 2) covering response categories (c) and (d), *At risk/need help* (score = 1) covering response categories (e) and (f) and *Unable* (score = 0) covering response category (g) [36]. The present version of the ADL-Q can also be used to measure the person’s perceived satisfaction with the quality of performance for each of the 47 ADL tasks, using a four-point ordinal satisfaction scale: 4 = ‘very satisfied’, 3 = ‘satisfied’, 2 = ‘dissatisfied’ and 1 = ‘very dissatisfied’ [36]. ADL-Q satisfaction measures are also generated based on the Rasch measurement methods [36]. ADL-Q performance measures have demonstrated sensitivity to change, when applied in persons with rheumatoid arthritis [2].

#### Occupational Balance Questionnaire

OBQ is an 11-item questionnaire evaluating occupational balance of individuals and groups. Occupational balance is defined as *“the experience of having the right amount of occupations and the right variation between occupations, including work, leisure, rest and sleep”* [38]. In OBQ11, the participants report their perceived occupational balance for each of 11 items, using a four-response category scale from 0 = ‘completely disagree’ to 3 = ‘completely agree’. Scores are summed into a total score ranging from 0 to 33, with 33 representing complete occupational balance. OBQ11 has been examined for internal construct validity in a general population using the Rasch measurement theory [37], but not yet in clinical samples.

#### Client-weighted problems

To complete the investigation on how, from the participants’ point of view, engagement in ADL task performance contribute to well-being, and how the participants experienced changes, five questions (CWP) (Additional file 2) were constructed specifically for this study, e.g.: “*How big a problem is it for you, that your chronic condition(s) affects your possibilities to perform and participate in daily tasks in and around your home (e.g. shopping, cleaning, doing laundry, transport)?*”. The questions were related to the participant’s identified problems and perceived need for help and hopes for the future. The perceived weight was scored on an 11-point numeric scale ranging from ‘0’ representing “*not at all*” to ‘10’ representing “*to a high extent*”.

### Accessible information on usual occupational therapy

Decisions on needed information on usual occupational therapy was structured by the MRC guidance [39], and inspired by Erlen et al. [40] and Hoffmann et al. [41]. Identification of the specific aspects of information was guided by several hypotheses on mechanisms of action in the ABLE 2.0 intervention program. Aspects included *dose* (duration of intervention, number of visits, length of visits), *evaluation of ADL ability* (use of standardised instruments, self-report and/or observation), *goal setting* (whether goals were formulated, how goals were negotiated), *content of treatment phase* (applied approaches including practicing performance of ADL tasks, counselling, focus on occupation/body functions/environment, involvement of home carer or relative), *referral services* (e.g. social services, group exercises or peer support groups) and *programmatic and/or clinical changes during trial* (changes applied based on e.g. new guidelines or participation in specialised courses) [40].

The monitoring method was the investigation of routinely collected records of participants receiving usual occupational therapy in the ABLE pilot (*n* = 10). A study-specific schedule for registering data on the predefined aspects of information was developed. Data collection was conducted by the primary investigator and a person from the municipal Rehabilitation Unit, specialised in client records and knowledgeable about rehabilitation practices in the municipality, but not otherwise involved in the study [40]. Progression criterion was access to information on the predefined aspects of usual occupational therapy in 80% of the participants.

### Procedures

Following inclusion, a letter was sent to the participants, containing written information on the ABLE pilot study, informed consent form and questionnaires. A baseline home visit by an assessor was scheduled within seven weekdays from the inclusion and oral consent. At the visit, the participant was asked to hand in the signed informed consent form and the filled-in questionnaires. If the participant needed help to fill in any of these, the assessor offered and registered the need of help. Thereafter, observation-based evaluation of ADL ability using the AMPS [26, 27] was performed.

To minimise contamination between interventionists, ABLE 2.0 was delivered by OTs employed in Rehabilitation Unit areas West and East, whereas usual occupational therapy was delivered by OTs employed in Rehabilitation Unit areas South and North. The OTs had rare contact across areas, and ABLE OTs were informed not to share information of any kind on ABLE 2.0 with their colleges. Furthermore, the OTs delivering ABLE 2.0 did not deliver usual occupational therapy. Still, to be able to randomise at an individual level, both the ABLE OTs and the usual occupational therapy OTs delivered interventions in all four geographical areas, depending on the outcome of the randomisation.

External assessors were masked on allocation to intervention at post-intervention and follow-up.

### Sample size

Based on the study aims, sample size calculation was not required [42, 43]. Rather, the number of participants was based on representativity related to the target study population, and a sample size large enough to provide useful information about the aspects of the study [43]. Hence, it was decided to include 20 participants.

### Data analyses

Data were analysed using IBM SPSS Statistics, version 25. Nominal and ordinal data were reported as number and percentage. Continuous variables were reported as mean and standard deviation (SD), provided that data were normally distributed. Ordinal data and data with lack of normal distribution were presented based on median and range, and nominal data based on percentages. Participant demographic data on age, gender, diagnosis, civic status, job situation, educational level, ADL ability, occupational balance and self-reported general health were presented in a table.

### Recruitment, retention and randomisation

Data on recruitment and retention, including number of participants recruited and retention rate, and on randomisation procedures, including flow of participants in relation to randomisation, were presented in flowcharts.

### Adherent delivery of ABLE 2.0

Data in registration forms concerning what and how much was delivered, deviations from the intervention manual, work on goal setting, evaluation of ADL ability, unintended side effects and practical and/or organizational facilitators and barriers were summarised and presented in a table, and supported by quotes presented in text.

### Appropriateness of outcome measurements

Number of relevantly and fully answered ADL-Q, OBQ11 and CWP questionnaires were reported in numbers and percentages.

### Accessible information on usual occupational therapy

Overview on whether information on predefined aspects of usual occupational therapy was accessible or not was provided in a table. Furthermore, it was described if the quality of the information related to goal setting and content of usual occupational therapy was sufficient to be compared to similar types of information gathered during the ABLE intervention.

## Results

### The COVID-19 pandemic

Due to the COVID-19 pandemic, the ABLE pilot study was truncated on March 12th, 2020. Consequently, an evaluation was performed to determine the extent to which the collected data was sufficient to address the study aims. Additional actions were launched where possible. Data related to monitoring recruitment and randomisation procedures were judged to be sufficient. Information on retention was limited, and rates could not be determined. Data on adherence to program was limited with no opportunity to gather further data. Thus, results of adherence to intervention program was based on information from registration forms related to two completed and three interrupted ABLE interventions. Data on appropriateness of outcome measurements was limited, based on baseline evaluations of 13 participants. Due to the limited data, a supplementary group interview with assessors on their experiences from baseline assessments was conducted. As the number of baseline ADL-Q data was insufficient for generating ADL-Q measures, they could not be reported. Results of information on usual occupational therapy were based on three completed cases. Therefore, information from client records on another seven clients, representative for the study sample and receiving usual occupational therapy interventions before the pilot study, were included. Despite the truncation of the study, it was concluded that the pilot data were sufficient to answer the majority of the study questions.

### Presentation of sample

Participant demographic data are presented in Table 1.

**Table 1:** Participant baseline characteristics (*n* = 13)

	Total (***n*** = 13)	ABLE 2.0 (***n*** = 6)	UOT (***n*** = 7)
**Gender**: Female, n (%)	10 (77)	4 (67)	6 (86)
**Age**: Median (range)	81 (46–99)	82 (73–93)	81 (46–99)
**Diagnosis**: n (%)			
Neurological	6 (46)	3 (50)	3 (43)
Medical	2 (15)	0 (0)	2 (33)
Musculoskeletal	5 (38)	3 (50)	2 (33)
**Civic status**: n (%)			
Living alone	6 (46)	3 (50)	3 (50)
Living with partner	6 (46)	2 (33)	4 (57)
Living with partner and children	1 (8)	1 (17)	0 (0)
**Job situation**: n (%)			
Senior citizen or early retirement	13 (100)	6 (100)	7 (100)
**Educational level**: n (%)			
Lower level education ^a^	10 (77)	4 (67)	6 (86)
Higher level education ^b^	3 (23)	2 (33)	1 (14)
**SF-1 of SF-36**: Self-reported general heath: median (range)	4 (1–5)	4 (1–4)	4 (3–5)
**AMPS ADL motor ability**: mean (SD)	0.92 (0.36)	0.83 (0.27)	1.0 (0.42)
**AMPS ADL process ability**: mean (SD)	0.87 (0.29)	0.93 (0.34)	0.81 (0.26)
**Occupational Balance Questionnaire**: median (range)	22.5 (7–33)	23.50 (20–33)	19 (7–31)

Neurological: parkinsonism, stroke, multiple sclerosis

Medical: chronic obstructive pulmonary disease, cardiovascular disease

Musculoskeletal: osteoarthritis, back/neck pain, rheumatoid arthritis, shoulder pain

*UOT* usual occupational therapy

^a^ Collapse of three subgroups (primary school, vocational education, short higher education)

^b^ Collapse of two subgroups (medium-term higher education, higher education)

A total of *n* = 37 persons with chronic conditions were assessed for eligibility, and *n* = 18 were enrolled. Demographic data indicated variation across diagnoses, age, gender, civic status and educational level. Baseline mean AMPS ADL motor ability measures were below competence cut-off (< 2.0 logits) in both the ABLE and usual occupational therapy group, indicating physical effort, fatigue and/or clumsiness during ADL task performance. Also, baseline mean AMPS ADL process ability measures were below competence cut-off (< 1.0 logit), suggesting ineffective use of time, space and objects, safety risk and potential need for assistance in everyday life [26, 27].

Below, results are presented in relation to the specific aims of the pilot study.

### Recruitment and retention

**Fig. 2:**
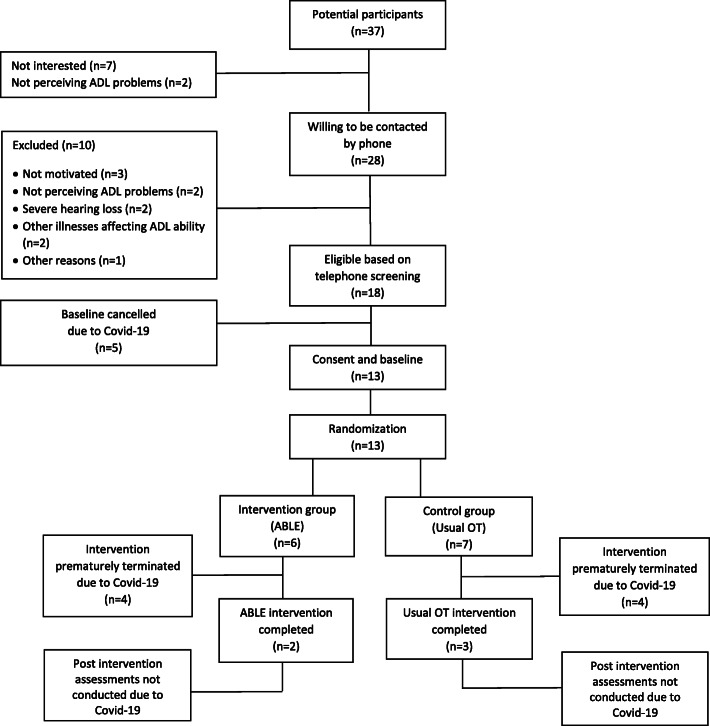
CONSORT diagram for pilot and feasibility trials: the ABLE 2.0 pilot

Of 37 potential participants contacted, 18 agreed to participate, resulting in an inclusion of 48.6%. Of these, 13 participants went through baseline evaluations before study was truncated (Fig. 2). Seven of the 13 participants needed help filling out the questionnaires. One participant had a limited use of the scale on ADL-Q performance (a score of 6 in 45 of 47 items). She explained her scores by saying: “*I can perform all tasks, but I tend to not get it done*”. Thus, even though she received daily assistance from spouse to initiate her task performances, she rated her ability to perform the tasks as independent and competent. Furthermore, on the CWP questionnaire she reversed her answers completely, when the assessor gave her further information on the scale.

In relation to retention, no participants dropped out of the study during the active data collection period.

### Randomisation procedure

None of the 18 eligible participants refused randomisation. In five cases, further procedures were interrupted due to the COVID-19 pandemic causing lockdown in the municipality. Hence, 13 participants were randomised, with six participants allocated to the ABLE arm and seven to usual occupational therapy arm. All 13 participants stayed in their allocated program until the lockdown (Fig. 2).

### Adherent delivery of ABLE 2.0

#### Sessions delivered, instruments applied, intervention components implemented, and time used

Two participants completed ABLE 2.0 with a minimum of five sessions, and one participant completed ABLE 2.0 except the final session. Another participant completed sessions 1 and 2. In all four cases, evaluation of ADL ability (AMPS and ADL-I) was conducted, and GAS was used for negotiating and setting goals in accordance with the manual. One more participant completed session 1 and went through ADL evaluations (Fig. 2). Finally, one randomised participant did not receive any sessions, before study ending. The two participants completing ABLE 2.0, also went through ADL re-evaluation (AMPS and ADL-I) in the final session as prescribed in the manual.

Eight of the nine potential intervention components to be applied during sessions 3–7 were applied across participants receiving ABLE 2.0 (Table 2).

**Table 2:** Frequency of implemented intervention components throughout sessions 3–7 in ABLE 2.0

ABLE 2.0 intervention components^a^ organised by PEO^b^	Frequency
P1: Changing habits related to task performance	3
P2: Changing attitude	3
P3: Plan, prioritise and reject	0
E1: Changing the physical environment	1
E2: Changing the social environment	1
E3: Use of tools, technology and/or helping aids	3
E4: Referring to other relevant services and opportunities	2
O1: Dividing the task into minor steps/distributing the task performance over longer time	1
O2: Simplifying the process/simplifying the task	1
Homework between sessions^c^	3

^a^ Based on *n* = 3 participants who completed the minimum of five intervention sessions

^b^ *Abbreviations*: *P* Person, *E* Environment, *O* Occupation

^c^ Homework between session was applied in all three cases; examples were taking the bus with a friend, practice preparing lunch in smaller parts with rests in between, and practice using cordless vacuum cleaner

The median number of minutes spent at sessions delivered face-to-face varied between 27 and 135 minutes with a tendency to spend more time on the first (median = 93 min) and final sessions (median = 72 min) involving ADL evaluations.

In the usual occupational therapy group, three participants completed their intervention process. Another participant had the intervention process interrupted after one visit, and three participants did not enter the usual occupational therapy intervention.

#### Deviations from the manual

The OTs reported no deviations from the manual, only adjustments within the inherent flexibility of the program. Instruments and models were applied according to the manual.

#### Goal setting

Goals were negotiated for all participants completing session 2 (*n* = 4). Two participants each defined two goals, and two participants each defined one goal. The OTs’ satisfaction with delivering session 2 was high (median = 4.5; range: 3 to 5), whereas the OTs experience on how the dialogue on goal setting worked was somewhat lower (median = 3; range: 2 to 5) (Table 3).

**Table 3:** Pilot aspects related to delivery of the ABLE 2.0 (registrations^a^ from OTs (*n* = 3) and participants (*n* = 6))

		Session 1	Session 2	Sessions 3–7	Final session
**Number of OT registrations:** n (%)		5 (83) ^b^	4 (100)	6 (100)	2 (67) ^b^
**Session 1**	The session gave me knowledge on which ADL tasks and skills are problematic: median (range)	4.0 (3–5)			
	The session clarified focus (ADL tasks and skills) for intervention: median (range)	4.0 (3–5)			
	The participant and I established a good basis for further cooperation: median (range)	4.0 (3–5)			
**Session 2**	The dialogue on discrepancy worked well: median (range)		4.0 (3–5)		
	The dialogue on goal setting worked well: median (range)		3.0 (2–5)		
	The dialogue on reasons for ADL problems worked well: median (range)		4.0 (4)		
**Sessions 3–7**	The session contributed to goal attainment: median (range)			3.0 (2–4)	
	The participant and I had a good cooperation on finding new strategies: median (range)			4.0 (3–5)	
	The participant was willing to try new strategies: median (range)			4.0 (2–5)	
**Final session**	The intervention overall contributed to goal attainment: (range)				(3)
	The intervention overall contributed to better ADL ability: (range)				(4)
	I believe client will carry on using new strategies: (range)				(3, 4)
**Questions asked on all sessions**	Confidence in delivering: median (range)	4.0 (4–5)	4.5 (3–5)	4.0 (3–5)	(4, 5)
	OT engagement: median (range)	4.5 (4–5)	5.0 (4–5)	4.0 (3–5)	(5)
	Involvement of client: median (range)	3.5 (3–4)	4.0 (4)	4.0 (3–4)	(4, 5)
	Perceived meaningfulness: median (range)	4.0 (3–4)	4.5 (3–5)	4.0 (2–5)	(4, 5)
	Perceived client meaningfulness: median (range)	3.5 (3–4)	3.5 (3–5)	3.5 (3–4)	(4, 5)
	Perceived satisfaction on delivery: median (range)	3.5 (2–4)	3.5 (3–5)	4.0 (2–5)	(4, 5)
	Perceived client satisfaction: median (range)	4.0 (3–5)	3.5 (3–5)	4.0 (3–5)	(4, 5)
**Number of participant registrations:** n (%)		5 (83)	4 (100)	6 (100)	2 (67)
**Session 1**	ADL-I and AMPS gave me new knowledge on my ADL problems: median (range)	2.0 (2–3)			
	ADL-I and AMPS clarified focus for intervention: median (range)	4.0 (2–4)			
	OT and I established a good basis for further cooperation: median (range)	4.0 (3–5)			
	I can see a purpose in participating in program: median (range)	4.0 (2–5)			
**Session 2**	I liked the work on goal setting: median (range)		4.0 (4)		
	It was relevant to talk about reasons for my ADL problems: median (range)		4.0 (3–4)		
	I can see a purpose in participating in program: median (range)		4.0 (4)		
**Sessions 3**–**7**	Session contributed to goal attainment: median (range)			3.5 (3–5)	
	I have at this point attained my goals: median (range)			3.0 (2–3)	
	I can see a purpose in participating in program: median (range)			4.0 (3–5)	
**Final session**	Intervention overall contributed to goal attainment: (range)				(3, 4)
	Intervention overall contributed to better ADL ability: (range)				(3, 4)
	I will carry on using the new strategies: (range)				(3, 4)
**Questions asked on all session**	I felt informed: median (range)	4.0 (3–5)	4.0 (4)	3.5 (3–5)	(3, 4)
	I felt involved: median (range)	4.0 (4–5)	4.0 (3–4)	4.0 (3–5)	(4)
	Session was meaningful to me: median (range)	4.0 (3–5)	4.0 (4)	4.0 (3–5)	(4, 5)
	Session was satisfactory to me: median (range)	4.0 (3–5)	4.0 (4)	4.0 (4–5)	(4)

^a^ Scored using Likert scales from 1–5; 1 = very low degree, 2 = low degree, 3 = fair degree, 4 = high degree and 5 = very high degree

^b^ One registration form was not completed

The OTs perceived some challenges related to goal setting: “*difficult to guide the participant on grading the goals*”; “*participant found it difficult to understand the scale*”; and “*it was difficult to explain GAS*”. Still, the participants all reported that they highly appreciated working with goal setting (median = 4).

#### Confidence, engagement, involvement of participant, meaningfulness and satisfaction with ABLE 2.0

The OTs’ confidence in delivering ABLE 2.0 was high, and they felt highly engaged during the sessions (Table 3). Degree of participant involvement was high, with similar scores from OTs and participants. Participants and OTs found the content of the sessions highly meaningful and satisfactory.

#### Unintended side effects

OTs registrated a few examples of positive side effects: “*Based on the ADL task performance during the session, the participant was more able to describe the experienced problem related to the task*”; “the *participant seemed more motivated* [at the end of session 2]”.

#### Practical and/or organizational facilitators and barriers

There were no registrations of problems related to access to needed helping aids.

### Appropriateness of outcome measurements

At baseline, four participants (30.7%) completed the performance ratings of the ADL-Q. In contrast, only two participants (15.4%) completed the satisfaction ratings of the ADL-Q. The OBQ11 assessments at baseline was completed by twelve participants (92.3%). Finally, all participants (100%) completed the CWP questionnaire at baseline. Thus, the progression criterion of 80% completely answered questionnaires was met in OBQ11 and CWP, but not in ADL-Q.

Seven participants needed assistance to fill in the questionnaires, one due to limited vision, another six for reasons like “*lack of overview*”, *“overwhelming*”, “*lack of energy*”, “*receiving the questionnaires only the day before the meeting [baseline assessment]*” and “*not understanding a term* [occupational balance]”. Two of these seven participants needing help filling in the questionnaires, received only minor assistance (less than 10% of the items) to complete.

### Information on usual occupational therapy

Table 4 presents information on which of the predefined aspects of usual occupational therapy information was accessible.

**Table 4:** Information on usual occupational therapy, accessible in client records (*n* = 10)

Aspect	Prespecified information	Access to information
		Yes
**Dose**	Duration of intervention in days	10
	Number of visits	10
	Duration of visits in minutes	0^a^
**Evaluation of ADL ability**	Applied methods^b^	9
**Goal setting**	Whether goals were formulated	9
	How goals were negotiated	9
**Content of treatment phase**	Applied approaches^c^	10
**Referral services**		10
**Programmatic and/or clinical changes**^d^		0

^a^Scheduled time was accessible

^b^Use of standardised instruments; use of observation; use of self-report

^c^Practicing performance of ADL tasks; counselling; focus on occupation/body functions/environment; involvement of home carer or relative

^d^Changes applied based on e.g. new guidelines or participation in specialised courses

The quality of the information related to goal setting and content of usual occupational therapy was assessed to be sufficient for comparison to similar types of information gathered during the ABLE intervention.

## Discussion

This pilot study evaluated the remaining feasibility aspects of the ABLE 2.0 intervention program in terms of design, conduct and processes of an outcome trial, including recruitment, randomisation, adherence, appropriateness of outcome measurements and access to information on usual occupational therapy. The results indicated that the procedures for recruitment and randomisation were feasible and that ABLE 2.0 was delivered according to the manual and with engagement. OTs were overall satisfied delivering the ABLE intervention. Moreover, adherence was sufficient since the minimum number of sessions, the mandatory assessments and intervention components for good quality of ABLE 2.0 intervention delivery, were applied by the OTs. Additionally, it was possible to extract almost all the desired information on usual occupational therapy from the client records. Concerning the outcome measurements, the application of ADL-Q in this client population was associated with challenges, whereas the OBQ11 and CWP were eligible.

The revised procedures on recruitment enabled inclusion, as almost half of the persons referred agreed to participate. This differs from the results of the former feasibility study [23], suggesting the revised procedures are recommendable in a future trial. Considering the challenges related to answering the questionnaires, and the inclusion criteria on ‘*being able to understand and relevantly answer a questionnaire’*, we recognise that we are dealing with a population that might be challenged on this criterion. Striving at recruiting persons who seem to match the aims of the intervention and a sample as less biased as possible [44], it is suggested to reduce the amount of questionnaires rather than exclude persons being on the edge of this criteria. Furthermore, we suggest asking potential future participants if they feel confident in answering questionnaires.

In this study, one person, referred for the study, needed help filling in the questionnaires due to limited vision, and another two persons, referred, could not be provided with information on the study due to limited hearing. Hence, their sensory losses introduced a risk to quality of data, preventing them from participation in the study. Accordingly, the exclusion criteria on ‘*language barriers*’ should be adjusted to ‘*communication barriers*’. Another three persons, referred, were not included due to lack of motivation. The legislation in Denmark prescribes that persons, who apply for home care to assist with household chores, instead as a standard procedure are referred to reablement, a time-limited intervention provided in people’s homes to support reacquisition of skills to manage their household chores [45]. Being referred to intervention rather than receiving the requested home care, may have resulted in a higher number of potential participants at entrance of the pilot study, who not all were motivated for participating in the program. Furthermore, research indicate that elderly persons who are frail and have decreased health are more difficult to recruit into research [44, 46], as reflected in the progression criteria of 50% on recruitment in this pilot study. Knowing that differences between participants and non-participants might bias the results of a future RCT and decrease external validity [44], much attention should be paid on recruitment in a future trial.

The challenges on adherent delivery of the first version of ABLE intervention program revealed in the ABLE feasibility study was related to application of AMPS and goal setting. In the present study involving ABLE 2.0, all instruments were applied according to the manual, and only few adjustments were made delivering the sessions, all within the frame of the program. The results indicate that the revisions of the manual and the tailored course for the OTs overall were efficient. In addition, it is appropriate to emphasise, with reference to the MRC’s guidance [47], that some flexibility in the intervention program should be allowed, as interventions may work better if adaptation is acceptable. Thus, the inherent flexibility in ABLE 2.0 is regarded a strength. Results of the present pilot study related to dose (sessions delivered, intervention components implemented and time use) was quite in line with the positive results from the ABLE feasibility study on the same aspects. Hence, the minimum of five sessions should be maintained.

The biggest challenge on outcome measurements in the ABLE 2.0 pilot was related to answering the ADL-Q performance and satisfaction scales, as only 4 of 13 scored the performance scale, and two of 13 scored the satisfaction scale. Fortunately, we learned from the former feasibility study [23] that the interview-based equivalent, ADL-I, is feasible in this population. Hence, the use of ADL-I seems more appropriate to use in this population as it likely provides more complete datasets. Previous research has shown that measures of ADL ability is dependent on the methods applied with questionnaire and interview yielding different but related information about ADL ability [2]. The pattern is a higher self-reported ADL ability based on questionnaire compared with interview [2]. Thus, it is recommended to replace the ADL-Q with the ADL-I, evaluating self-reported ADL ability in terms of performance and satisfaction in a future trial. Furthermore, this will ease the participants´ burden related to answering questionnaires.

Thorough information on usual occupational therapy is critical for investigating effectiveness of the ABLE 2.0 intervention program [20, 41, 48]. Hoffmann et al. [41] suggest describing usual care in a trial with the same level of detail as in the intervention group. But usual care is by nature a dynamic phenomenon. Therefore, it is unlikely that all participants in a control group will receive the same usual care, and furthermore, usual care typically reflects locally adapted practices and may vary at different time points during a trial [40, 49]. Hence, description of usual occupational therapy, based on retrospective investigation on what was delivered to participants receiving usual occupational therapy, should be optimal in a future trial. Information on actual duration of each visit could be requested documented in a future RCT, providing data that are comparable to information gathered during the ABLE intervention. Due to lack of accessibility to information on programmatic and/or clinical changes in the client records in the municipality, it is recommended to conduct short and focused interviews on this aspect, with OTs delivering usual occupational therapy, after the intervention period in a future RCT. Also, it is recommended that data collection on usual occupational therapy is conducted by research staff assisted by a person from the Rehabilitation Unit in the municipality, familiar with clinical practice and client records, to extract information on all possible aspects. Finally, it is recommended to maintain the study-specific schedule developed for this pilot, to collect consistent data on usual occupational therapy interventions.

## Conclusions

This pilot study has provided useful information on important aspects related to evaluating the ABLE 2.0 intervention program. Adding the results of this study to the results of the previous feasibility study, and following the recommendations of the MRC guidance on developing and evaluating complex health interventions, progressing to a full-scale RCT including evaluation of effectiveness, processes and economy of the ABLE 2.0 program is recommendable. A limitation of the study is the incomplete dataset, caused by the COVID-19 pandemic prematurely terminating the study and resulting in weaker evidence on some pilot aspects, primarily on adherence to ABLE 2.0 and appropriateness of outcome measurements. There are important findings though, that the procedures on recruitment and randomisation were effective and that it was possible to recruit a sample representing the population being target group of the ABLE intervention. Further, for planning a future trial, it is important to know that the ABLE intervention was delivered according to the manual and that the first five persons included completed the intervention sessions and stayed in the program.

## Supplementary Information

**Additional file 1.** Description of instruments integrated in the ABLE 2.0 Intervention Program.

**Additional file 2.** Client Weighted Problems (CWP).

**Additional file 3.** CONSORT 2010 checklist of information to include when reporting a pilot or feasibility trial*.

## Data Availability

The datasets used and/or analysed during the current study are available from the corresponding author on reasonable request.

## Abbreviations

ADL: Activities of daily living
ABLE: A Better everyday LifE
MRC: Medical Research Council
OT: Occupational therapist
RCT: Randomised controlled trial
AMPS: Assessment of Motor and Process Skills
OTIPM: Occupational Therapy Intervention Process Model
GAS: Goal Attainment Scaling
ADL-Q: Activities of Daily Living Questionnaire
ADL-I: Activities of Daily Living Interview
CWP: Client-Weighted-Problems Questionnaire
OBQ11: Occupational Balance Questionnaire
SF-36: MOS 36-Item Short Form Survey Instrument
SD: Standard deviation

## References

[1] Hvidberg MF, Johnsen SP, Davidsen M, Ehlers L (2019). A nationwide study of prevalence rates and characteristics of 199 chronic conditions in Denmark. PharmacoEconomics - Open.

[2] Wæhrens EE, Bliddal H, Danneskiold-Samsøe B, Lund H, Fisher AG (2012). Differences between questionnaire-and interview-based measures of activities of daily living (ADL) ability and their association with observed ADL ability in women with rheumatoid arthritis, knee osteoarthritis, and fibromyalgia. Scand J Rheumatol.

[3] Nielsen KT, Wæhrens EE (2015). Occupational therapy evaluation: use of self-report and/or observation?. Scand J Occup Ther.

[4] Bendixen HJ, Wæhrens EE, Wilcke JT, Sørensen LV (2014). Self-reported quality of ADL task performance among patients with COPD exacerbations. Scand J Occup Ther.

[5] Lindahl-Jacobsen L, Hansen DG, Wæhrens EE, la Cour K, Søndergaard J (2015). Performance of activities of daily living among hospitalized cancer patients. Scand J Occup Ther.

[6] Daving Y, Claesson L, Sunnerhagen KS (2009). Agreement in activities of daily living performance after stroke in a postal questionnaire and interview of community-living persons. Acta Neurol Scand.

[7] Hariz GM, Forsgren L (2011). Activities of daily living and quality of life in persons with newly diagnosed Parkinson’s disease according to subtype of disease, and in comparison to healthy controls. Acta Neurol Scand.

[8] Norberg EB, Boman K, Löfgren B (2008). Activities of daily living for old persons in primary health care with chronic heart failure. Scand J Caring Sci.

[9] Månsson Lexell E, Iwarsson S, Lexell J (2006). The complexity of daily occupations in multiple sclerosis. Scand J Occup Ther.

[10] Goodman RA, Posner SF, Huang ES, Parekh AK, Koh HK (2013). Defining and measuring chronic conditions: imperatives for research, policy, program, and practice. Prev Chronic Dis.

[11] Wæhrens EE. Almindelig daglig levevis. Munksgaard, København: ADL; 2015.

[12] Avlund K, Schultz Larsen K, Kreiner S (1993). The measurement of instrumental ADL: Content validity and construct validity. Aging Clin Exp Res.

[13] Steultjens E, Dekker J, Bouter L, Leemrijse C, van den Ende C (2005). Evidence of the efficacy of occupational therapy in different conditions: an overview of systematic reviews. Clin Rehabil.

[14] Hand C, Law M, McColl MA (2011). Occupational therapy interventions for chronic diseases: a scoping review. Am J Occup Ther.

[15] Guidetti S, Ranner M, Tham K, Andersson M, Ytterberg C, Von Koch L (2015). A “client-centred activities of daily living” intervention for persons with stroke: one-year follow-up of a randomized controlled trial. J Rehabil Med.

[16] Nielsen TL, Petersen KS, Nielsen CV, Strøm J, Ehlers MM, Bjerrum M (2017). What are the short-term and long-term effects of occupation-focused and occupation-based occupational therapy in the home on older adults’ occupational performance? A systematic review. Scand J Occup Ther.

[17] Sturkenboom IHWM, Graff MJL, Hendriks JCM, Veenhuizen Y, Munneke M, Bloem BR, Nijhuis-van der Sanden M, OTiP study group (2014). Efficacy of occupational therapy for patients with Parkinson’s disease: a randomised controlled trial. Lancet Neurol.

[18] Nielsen KT, Klokker L, Wæhrens EE. Self-reported quality of activities of daily living (ADL) task performance in four diagnostic groups with chronic conditions. International Journal of Therapy and Rehabilitation 2021;28(4):1–10. 10.12968/ijtr.2020.0025.

[19] Nielsen KT. Occupational therapy for persons living with chronic conditions - development and feasibility of the ABLE program. Odense: University of Southern Denmark; 2018.

[20] Craig P, Dieppe P, Macintyre S, Mitchie S, Nazareth I, Petticrew M (2008). Developing and evaluating complex interventions: the new Medical Research Council guidance. BMJ..

[21] Nielsen KT, Klokker L, Guidetti S, Wæhrens EE. Identifying, organizing and prioritizing ideas on how to enhance ADL ability. Scand J Occup Ther. 2019;26(5):382–293. 10.1080/11038128.2018.1424235. 10.1080/11038128.2018.1424235 29322869

[22] Hoffmann T, Bennett S, Del Mar C (2010). Evidence-based practice across the health professions.

[23] Nielsen KT, Guidetti S, von Bülow C, Klokker L, Wæhrens EE. Feasibility of ABLE 1.0 – a program aiming at enhancing the ability to perform activities of daily living in persons with chronic conditions. Pilot Feasibility Stud. 2021;7(1):1–15. 10.1186/s40814-021-00790-7 PMC7891027 33602338

[24] O’Cathain A, Hoddinott P, Lewin S, Thomas KJ, Young B, Adamson J (2015). Maximising the impact of qualitative research in feasibility studies for randomised controlled trials: guidance for researchers. Pilot Feasibility Stud.

[25] Fletcher A, Jamal F, Moore G, Evans RE, Murphy S, Bonell C (2016). Realist complex intervention science: applying realist principles across all phases of the Medical Research Council framework for developing and evaluating complex interventions. Evaluation..

[26] Fisher AG, Jones KB (2012). Assessment of motor and process skills. Volume 1: development, standardization, and administration manual.

[27] Fisher AG, Jones KB (2012). Assessment of motor and process skills. Volume 2: user manual.

[28] Fisher AG, Marterella A (2019). Powerful practice: a model for authentic occupational therapy.

[29] Strong S, Rigby P, Stewart D, Law M, Letts L, Cooper B (1999). Application of the Person-Environment-Occupation Model: a practical tool. Can J Occup Ther.

[30] Wæhrens EE, Nielsen KT. ADL-Interview (ADL-I). Klinisk version 1.0 - Introduktion, ADL-I og administration. Copenhagen: ACE; 2020.

[31] Kiresuk TJ, Smith A, Cardillo JE (1994). Goal Attainment Scaling: applications, theory, and measurement.

[32] Krasny-Pacini A, Hiebel J, Pauly F, Godon S, Chevignard M (2013). Goal Attainment Scaling in rehabilitation: a literature-based update. Ann Phys Rehabil Med.

[33] Bowen DJ, Kreuter M, Spring B, Cofta-Woerpel L, Linnan L, Weiner D, Bakken S, Kaplan CP, Squiers L, Fabrizio C, Fernandez M (2009). How we design feasibility studies. Am J Prev Med.

[34] Charlesworth G, Burnell K, Hoe J, Orrell M, Russell I. Acceptance checklist for clinical effectiveness pilot trials: a systematic approach. BMC Med Res Methodol. 2013;13(78):1–7. 10.1186/1471-2288-13-78 PMC3702517 23758922

[35] Wild D, Alyson G, Mona M, Sonya E, Sandra M, Verjee-Lorenz A (2005). Principles of good practice for the translation and cultural adaptation process for patient-reported outcomes (PRO) measures. Value Health.

[36] Wæhrens EE (2010). Measuring quality of occupational performance based on self-report and observation. Development and validation of instruments to evaluate ADL task performance.

[37] Håkansson C, Wagman P, Hagell P (2019). Construct validity of a revised version of the Occupational Balance Questionnaire. Scand J Occup Ther.

[38] Wagman P, Håkansson C, Björklund A (2012). Occupational balance as used in occupational therapy: a concept analysis. Scand J Occup Ther.

[39] Moore G, Audrey S, Barker M, Bond L, Bonell C, Hardeman W (2014). Process evaluation of complex interventions. UK Med Res Counc Guid.

[40] Erlen JA, Tamres LK, Reynolds N, Golin CE, Rosen MI, Remien RH, Banderas JW, Schneiderman N, Wagner G, Bangsberg DR, Liu H (2015). Assessing usual care in clinical trials. West J Nurs Res.

[41] Hoffmann TC, Glasziou PP, Boutron I, Milne R, Perera R, Moher D (2014). Better reporting of interventions: template for intervention description and replication (TIDieR) checklist and guide. BMJ..

[42] Billingham SA, Whitehead AL, Julious SA (2013). An audit of sample sizes for pilot and feasibility trials being undertaken in the United Kingdom registered in the United Kingdom Clinical Research Network database. BMC Med Res Methodol.

[43] Thabane L, Ma J, Chu R, Cheng J, Ismaila A, Rios LP, Robson R, Thabane M, Giangregorio L, Goldsmith CH (2010). A tutorial on pilot studies: the what, why and how. BMC Med Res Methodol.

[44] Michelet M, Lund A, Sveen U (2014). Strategies to recruit and retain older adults in intervention studies: a quantitative comparative study. Arch Gerontol Geriatr.

[45] Aspinal F, Glasby J, Rostgaard T, Tuntland H, Westendorp RGJ (2016). New horizons: reablement - supporting older people towards independence. Age Ageing.

[46] Chatfield MD, Brayne CE, Matthews FE (2005). A systematic literature review of attrition between waves in longitudinal studies in the elderly shows a consistent pattern of dropout between differing studies. J Clin Epidemiol.

[47] Craig P, Dieppe P, Macintyre S, Michie S, Nazareth I, Petticrew M. Developing and evaluating complex interventions: the new Medical Research Council guidance. BMJ. 2008;337(7676). 10.1136/bmj.a1655 PMC2769032 18824488

[48] Boutron I, Moher D, Altman DG, Schulz KF, Ravaud P (2008). Extending the CONSORT statement to randomized trials of nonpharmacologic treatment: explanation and elaboration. Ann Intern Med.

[49] Yorganci E, Evans CJ, Johnson H, Barclay S, Murtagh FEM, Yi D, Gao W, Pickles A, Koffman J (2020). Understanding usual care in randomised controlled trials of complex interventions: a multi-method approach. Palliat Med.

