# Supplementary material for: Occupational therapy addressing the ability to perform activities of daily living among persons living with chronic conditions: a randomised controlled pilot study of ABLE 2.0
Source: Pilot Feasibility Stud. 2021 Jun 11;7:122. doi: 10.1186/s40814-021-00861-9 (PMC8192272; doi:10.1186/s40814-021-00861-9)
Supplement: Supplementary file 1 — Additional file 1. Description of instruments integrated in the ABLE 2.0 Intervention Program. [file 40814_2021_861_MOESM1_ESM.docx]

**Appendix A:** Description of instruments integrated in the ABLE 2.0 Intervention Program

# Instruments integrated in the ABLE 2.0 intervention program

## The ADL Interview

The ADL-Interview (ADL-I) is used in the first and last sessions in the ABLE 2.0 intervention program. Conducting the ADL-I at session 1 serve as a starting point for the collaboration between the occupational therapist (OT) and the client identifying which Activities of Daily Living (ADL) task performance problems to be focusing on and set goals for in the intervention. ADL-I is a standardised evaluation tool, used by OTs, to describe and measure the self-reported quality of ADL task performance (1,2), in terms of physical effort and/or fatigue, efficiency, safety and independence. In the ADL-I, the clients report their perceived ADL ability for each of 47 ADL items using the following seven response categories: (a) I perform the task independently without use of extra time or effort and without risk; (b) I perform the task independently but I use helping aids; (c) I perform the task independently but it takes me extra time; (d) I perform the task independently but I use extra effort/get tired; (e) I perform the task independently but there is a risk that I might injure myself; (f) I need assistance from someone but do participate; and (g) the task is performed by others for me – I cannot participate actively. The client can mark more than one response category if several apply to their performance of the specific ADL task (e.g. mark both c and d if they spend extra time and get tired) (1,2).

To create an overall linear measure of self-reported quality of ADL task performance (reported in log-odds probability units; logits), based on Rasch measurement methods, the mark given in the lowest response category on each task is re-scored using an ordinal rating scale from 0 to 3: C*ompetent* (score =3) covering response categories (a) and (b), *Using extra time/effort* (score=2) covering response categories (c) and (d), *At risk/need help* (score =1) covering response categories (e) and (f) and *Unable* (score = 0) covering response category (g) (1).

Moreover, ADL-I can be used to measure the client’s perceived satisfaction with the quality of performance for each of the 47 ADL tasks, using a four-point ordinal satisfaction scale: 4=‘very satisfied’, 3=‘satisfied’, 2=‘dissatisfied’ and 1=‘very dissatisfied’ (1). ADL-I satisfaction measures are also generated based on Rasch Measurement methods.

To measure change in self-reported quality of ADL task performance and satisfaction, the 47 ordinal quality of performance and satisfaction scores are transformed into overall linear (interval scale) measures of self-reported quality of ADL task performance and satisfaction, adjusted for the difficulty of the ADL tasks, based on Rasch measurement methods (1). The measures are expressed in logits (log-odds probability units) (1,3).

Previous studies indicate that ADL-I can be used to generate valid and reliable linear measures of self-reported quality of ADL task performance among persons living with chronic conditions (1,4,5), and furthermore, that the instrument is sensitive to change post-intervention in older persons receiving a home-based reablement program (6). According to the ADL-I manual (2), a difference of >0.64 logits (based on mean SD=1.28) indicates a clinically relevant difference in self-reported ADL task performance.

## The Assessment of Motor and Process Skills (AMPS)

The Assessment of Motor and process Skills (AMPS) (7,8) is also used in the first and last sessions in the ABLE 2.0. It is a standardised observation-based evaluation tool used by OTs to measure a person’s observed quality of ADL task performance in terms of physical effort and/or fatigue, efficiency, safety and independence. The person being evaluated chooses and performs at least two of the standardised ADL tasks that the person finds relevant and of appropriate challenge. During an AMPS evaluation, two domains of occupational performance are evaluated: motor skills (16 items) and process skills (20 items). After the observation, the quality of each skill is evaluated on a four-point ordinal scale according to the scoring criteria in the AMPS manual (8). The available AMPS software (9), based on Many-Faceted Rasch statistics, makes it possible to convert the ordinal raw scores into overall linear ADL motor and ADL process ability measures adjusted for task challenge, skill item difficulty and rater severity. The measures are expressed in logits (log-odds probability units) (7). ADL motor ability measures below the 2.0 logits competence cutoff indicate increased physical effort, fatigue and clumsiness during task performance and ADL process ability measures below the 1.0 logit competence cutoff indicate inefficient and potentially unsafe ADL task performance suggesting need for assistance in everyday life. Moreover, ADL ability measures below the 1.50 logit independence cut-off on the ADL motor scale and below the 1.00 logit independence cut-off on the ADL process scale indicate a likely need for assistance (7). Measures below the lower independence cut-offs of 1.00 and 0.70 logits for ADL motor and ADL process ability, respectively, mark a need for moderate/maximal assistance (10). Several studies support that the AMPS ability measures are reliable and valid among persons with chronic conditions (3,5,11–13). Furthermore, several studies reveal that the AMPS demonstrates sensitivity to change post-intervention (13–16). According to the AMPS manual (7) a difference of ≥ 0.30 logits on the ADL motor and/or ADL process scales defines a clinically relevant difference in ADL ability. AMPS can only be administered by calibrated assessors.

## Goal Attainment Scaling

Goal Attainment Scaling (GAS) (17,18) is used in session 2 in the ABLE 2.0 to facilitate the dialogue between the client and the OT in the process of setting client-centred, quantifiable goals and to measure improvements towards these goals. Hence the client is actively involved in defining goals and describing the levels of goal attainment. When a goal is being defined, measurable and observable indicators that can be used to evaluate the progress towards the goal (e.g., independence, duration and frequency). The level of goal attainment is described using an ordinal scale from −2 to +2. The actual level of performance is described at level −1, and the expected level is described at level 0. Level +1 and level +2 are descriptions of what the person will be able to, if he or she achieves more than expected. Level −2 describes the level, where the person achieves less than expected. A feasibility study (19) concluded that GAS was applicable among older adults with multiple chronic conditions living at home.

# References

1. Wæhrens EE. Measuring quality of occupational performance based on self-report and observation. Development and validation of instruments to evaluate ADL task performance. Sweden, Umeå: Department of Community Medicine and Rehabilitation, Umeå University; 2010.

2. Wæhrens EE, Nielsen KT. ADL-Interview (ADL-I). Klinisk version 1.0 - Introduktion, ADL-I og administration. ACE Copenhagen. 2020.

3. Wæhrens EE, Bliddal H, Danneskiold-Samsøe B, Lund H, Fisher AG. Differences between questionnaire-and interview-based measures of activities of daily living (ADL) ability and their association with observed ADL ability in women with rheumatoid arthritis, knee osteoarthritis, and fibromyalgia. Scand J Rheumatol. 2012;41(2):95–102.

4. Bendixen HJ, Wæhrens EE, Wilcke JT, Sørensen LV. Self-reported quality of ADL task performance among patients with COPD exacerbations. Scand J Occup Ther. 2014 Jul 21;21(4):313–20.

5. Nielsen KT, Wæhrens EE. Occupational therapy evaluation: Use of self-report and/or observation? Scand J Occup Ther. 2015;22(1):13–23.

6. Winkel A, Langberg H, Wæhrens EE. Reablement in a community setting. Disabil Rehabil. 2015;37(15):1347–52.

7. Fisher AG, Jones KB. Assessment of motor and process skills. Volume 1: Development, standardization, and administration manual. 7th ed. Fort Collins, Colorado, USA: Three Star Press; 2012.

8. Fisher AG, Jones KB. Assessment of motor and process skills. Volume 2: User manual. 7th ed. Fort Collins, Colorado, USA: Three Star Press; 2012.

9. OT Assessment Package (OTAP). Fort Cloons, Colorado, USA: Center for Innovative OT Solutions; 2016.

10. Merritt BK. Utilizing AMPS ability measures to predict level of community dependence. Scand J Occup Ther. 2010;17(1):70–6.

11. Moore K, Merritt B, Doble SE. ADL skill profiles across three psychiatric diagnoses. Scand J Occup Ther. 2010;17(1):77–85.

12. Von Bülow C, Amris K, La Cour K, Danneskiold-Samsøe B, Wæhrens EE. Ineffective ADL skills in women with fibromyalgia: a cross-sectional study. Scand J Occup Ther. 2016;23(5):391–7.

13. Wæhrens EE, Amris K, Fisher AG. Performance-based assessment of activities of daily living (ADL) ability among women with chronic widespread pain. Pain. 2010;150(3):535–41.

14. Graff MJL, Vernooij-Dassen MJM, Thijssen M, Dekker J, Hoefnagels WHL, Rikkert MGMO. Community based occupational therapy for patients with dementia and their care givers: Randomised controlled trial. Br Med J. 2006;333(7580):1196–9.

15. Nielsen KT, Guidetti S, Bülow C von, Klokker L, Wæhrens EE. Feasibility of ABLE 1.0 – a program aiming at enhancing the ability to perform activities of daily living in persons with chronic conditions. Pilot Feasibility Stud. 2021;7(52).

16. Ellegaard K, von Bülow C, Røpke A, Bartholdy C, Hansen IS, Rifbjerg-Madsen S, et al. Hand exercise for women with rheumatoid arthritis and decreased hand function: An exploratory randomized controlled trial. Arthritis Res Ther. 2019;21(1):1–9.

17. Kiresuk TJ, Smith A, Cardillo JE. Goal Attainment Scaling : Applications, theory, and measurement. Hillsdale, N.J.: L. Erlbaum Associates; 1994.

18. Krasny-Pacini A, Hiebel J, Pauly F, Godon S, Chevignard M. Goal Attainment Scaling in rehabilitation: A literature-based update. Ann Phys Rehabil Med. 2013;56(3):212–30.

19. Toto PE, Skidmore ER, Terhorst L, Rosen J, Weiner DK. Goal attainment scaling (GAS) in geriatric primary care: A feasibility study. Arch Gerontol Geriatr. 2015;60(1):16–21.
