# Supplementary material for: Occupational therapy addressing the ability to perform activities of daily living among persons living with chronic conditions: a randomised controlled pilot study of ABLE 2.0
Source: Pilot Feasibility Stud. 2021 Jun 11;7:122. doi: 10.1186/s40814-021-00861-9 (PMC8192272; doi:10.1186/s40814-021-00861-9)
Supplement: Supplementary file 2 — Additional file 2. Client Weighted Problems (CWP). [file 40814_2021_861_MOESM2_ESM.pdf]

Appendix B: Client Weighted Problems Questionnaire (CWP)

**Client-Weighted-Problems (CWP)**	
**Aspect**	**Questions to be weighted***
**Identified problems**	To what extent is it a problem for you, that your chronic condition(s) affects your possibilities to perform and participate in everyday activities in and around your home? (e.g. shopping, cleaning, doing laundry, transport)?
	To what extent is it a problem for you, that your chronic condition(s) affects your possibilities to participate in social activities with friends and family?
**Need for assistance**	To what extent do you need help accepting your chronic condition(s)?
	To what extent do you need help to better cope with your everyday activities (e.g. perform them more securely, efficiently, with less effort or more independently?
**Hope for the future**	To what extent does your chronic condition(s) affect your hope for the future?

*The perceived weight is scored on an 11-point ordinal scale ranging from '0' representing “not at all” to '10' representing ”to a high extent”
